# Impact of timing on the invasion of synthetic bacterial communities

**DOI:** 10.1093/ismejo/wrae220

**Published:** 2024-11-05

**Authors:** Keven D Dooley, Lucas P Henry, Joy Bergelson

**Affiliations:** Committee on Microbiology, University of Chicago, 924 East 57th Street, Chicago, IL 60637, United States; Center for Genomics and System Biology, Department of Biology, New York University, 12 Waverly Place, New York, NY 10003, United States; Center for Genomics and System Biology, Department of Biology, New York University, 12 Waverly Place, New York, NY 10003, United States; Department of Ecology and Evolution, University of Chicago, 1101 East 57th Street, Chicago, IL 60637, United States

**Keywords:** microbial ecology, community ecology, invasion ecology, synthetic bacterial community, transient community dynamics

## Abstract

Microbial communities regularly experience ecological invasions that can lead to changes in composition and function. Factors thought to impact susceptibility to invasions, such as diversity and resource use, vary over the course of community assembly. We used synthetic bacterial communities to evaluate the success and impact of invasions occurring at different times during the community assembly process. Fifteen distinct communities were subjected to each of three bacterial invaders at the initial assembly of the community (“initial invasion”), 24 h into community assembly (“early invasion”), when the community was still undergoing transient dynamics, and 7 days into community assembly (“late invasion”), once the community had settled into its final composition. Communities were passaged daily and characterized through sequencing after reaching a stable composition. Invasions often failed to persist over time, particularly in higher richness communities. However, invasions had their largest effect on composition when they occurred before a community had settled into a stable composition. We found instances where an invader was ultimately excluded yet had profound and long-lasting effects on invaded communities. Invasion outcome was positively associated with lower community richness and resource use efficiency by the community, which varied throughout assembly. Our results demonstrate that microbial communities experiencing transient community dynamics are more affected by, and in some instances prone to, invasion, a finding relevant to efforts to modify the composition of microbial communities.

## Introduction

Humans rely on microbial communities at both a personal level in the functions performed by our microbiome [1], as well as a societal level in the importance of plant microbiomes to agriculture [2] and microbial communities to industrial applications such as waste water treatment [3]. As such, many efforts are underway to design or modify microbial communities that generate a desired composition and functionality [4, 5]. Maintaining the composition–functionality link is a major challenge in our efforts to manipulate microbial communities to serve our own ends. As has often been observed, communities that perform as designed *in vitro* or in a host under well-controlled conditions frequently fail to maintain composition and function when introduced into a complex natural environment because they are invaded by other microbes [6, 7, 8]. Furthermore, beneficial probiotics fail to deliver benefits if they cannot invade and persist when introduced into the host microbiome [9, 10]. Both scenarios of failure reflect the complexities of ecological invasion, highlighting the importance of understanding when and why communities become vulnerable to invaders.

Invasion ecology concerns the establishment and impact of novel species in ecological communities. Ecologists have long recognized that higher diversity communities are often more resistant to invasion [11, 12, 13, 14, 15, 16, 17, 18]. This relationship has been attributed to two mechanisms. First, more diverse communities tend to saturate the available niche space and utilize resources more efficiently, resulting in a more productive community [13, 19, 20]. When this occurs, the resident community consumes the majority of available resources and leaves little niche space for an invader to occupy [18, 19]. However, the positive relationship between diversity and productivity cannot always explain protection against invasion [13, 17, 21], which may instead arise from a second mechanism, a sampling effect. Sampling effects occur because a more diverse community is more likely by chance alone to include a resident that interacts directly with an invader [14, 15, 20, 22, 23]. If the sampling effect is the dominant mechanism responsible for protection against invasion, then it is not diversity alone, but the strength (e.g. strong vs. weak) and nature (e.g. antagonism vs. facilitation) of specific interactions between residents and potential invaders that determine the invasibility of a community. For example, a diverse community is more likely to contain competitors that directly block an invader but is also more likely to include facilitative interactions that promote successful invasions [13]. Particularly for microbial communities, feedback between resource availability and community composition are rapid as species are lost and change in abundance. The dynamics of community assembly thus reshape the link between species interactions and resource use [24, 25]; such variation may affect the invasibility of assembling communities.

Here, we seek to experimentally disentangle the roles of community richness, resource use, and the assembly process in determining resistance to invasion. We posit that the timing of an invasion impacts its outcome due to changes in the diversity (richness) and function (resource use) of communities as they assemble, and reassemble, over time. We consider the “outcome” of an invasion as both the success/failure of the invader to persist in a community over time and the effect of the invader on the composition of the community. Our results demonstrate that microbial communities that are experiencing transient dynamics are more greatly impacted by invasion, both as a consequence of resource availability and the sampling effect.

## Materials and methods

### Bacterial isolates and reference genomes

All bacterial isolates were originally isolated from the leaves of wild or field-grown *Arabidopsis thaliana* in the midwestern states of the USA (IL, IN, MI). The isolate names, taxonomic information, and assembly information are presented in Supplementary Table 1. Genomes are published on the NCBI whole genome repository (accessions: PRJNA953780, PRJNA1073231).

### *Arabidopsis* leaf medium

*Arabidopsis thaliana* (KBS-Mac-74, accession 1741) plants were grown in the University of Chicago greenhouse from January to March 2020. Seeds were densely planted in 15-cell planting trays and thinned after germination to 4–5 plants per cell. Above-ground plant material was harvested just before development of inflorescence stems. Plant material was coarsely shredded by hand before adding 100 g to 400 mL of 10 mM MgSO_4_ and autoclaving for 55 min. After cooling to room temperature, the medium was filtered through 0.2-μm polyethersulfone membrane filters to maintain sterility and remove plant material. The medium was stored in the dark at 4°C. Before being used for culturing, the medium was diluted 1:10 in 10 mM MgSO_4_.

### Assembly and culturing of synthetic communities

Fresh bacterial stocks were prepared by first inoculating isolates into 1 mL of *Arabidopsis* leaf medium (ALM) shaking at 28°C and growing overnight. Next, 100 μL of these cultures was used to inoculate 5 mL of ALM shaking at 28°C. Once the cultures were visibly turbid, they were divided into 1-mL aliquots with sterile DMSO added to a final concentration of 7% as a cryoprotectant. Stocks were stored at −80°C. An aliquot of each stock was used to estimate bacterial cell density through colony counting on ALM plates. To initiate an experiment, stocks were diluted to densities determined by the target initial titer (1 × 10^6^ cells) of the community and the number of initial members. Diluted stocks were then combined into desired communities (see “Experimental design” below) and used to inoculate 600 μL of ALM in sterile 1-mL deep-well plates, in triplicate. Deep-well plates were covered with sterilized, loosely fitting plastic lids to allow air exchange. Plates were cultured in the dark at 28°C on high-speed orbital shakers capable of establishing a vortex in the deep-well plates to ensure that the cultures were well mixed. After 24 h, 6 μL of each culture was manually transferred by multichannel pipette into new plates containing 594 μL of fresh ALM. The new plates were immediately returned to the incubator and the day-old plates were stored at −80°C.

### Experimental design

**Figure 1:**
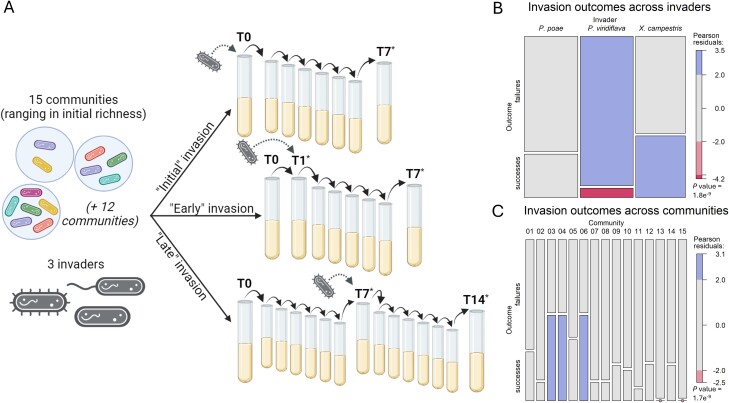
Experimental design and invasion outcomes across invaders and communities: (A) 15 bacterial communities were separately subjected to invasion by three bacterial isolates across three timing treatments. Across all treatments, passaging always occurred after 24 h and involved a 1:100 dilution into fresh media. In the “initial invasion” treatment, a given invader was added alongside the other community members at the time of community initiation (T0). In the “early invasion” treatment, a given community was assembled and passaged after 24 h (T1), before an invader was added immediately after passaging. And in the “late invasion” treatment, a given community was assembled and passaged for 7 days (T7) before an invader was added. Post-invasion, communities were passaged for 7 days and characterized using shallow short-read sequencing. Sequenced samples are indicated by (^*^). Figure created with BioRender.com. (B) Mosaic plot representing successful and failed invasions for each invader. Invasion outcome was defined as a “success” (bottom box) if the invader persisted over time or a “failure” (top box) if the invader was ultimately excluded. Chi-square test of independence shows invader identity was significantly associated with invasion success (*P* value 1.79e^−9^). (C) Mosaic plot representing successful and failed invasions in each community. Chi-square test of independence shows community identity was significantly associated with invasion success (*P* value 1.69e^−9^). Significantly over- or under-represented outcomes are highlighted. Pearson residuals >2 or <−2 indicate a count value >2 SD from the expectation.

To study invasion across multiple community contexts, we assembled a set of 15 synthetic bacterial communities from a pool of 48 bacterial strains, representing 24 genera (Supplementary Table 1). In previous work, we had characterized the dynamics of combinations of predefined sets of species in a nested design. That is, we considered single sets, pools of 2 sets, pools of 3 sets, etc. [26]. We selected a subset of these communities for use here. The communities that we selected had the properties that they settled into stable compositions that ranged in final richness and had compositions as disparate from each other as possible. Although we were relatively effective in reducing taxonomic redundancy between the lower initial richness communities, we could not avoid overlap between the communities of higher initial richness as these communities were composed of combinations of the less diverse communities. Given the high extinction rate in top-down experiments [26, 27], we chose to focus on these well-characterized communities as their consistency would allow us to observe the effect of invasion. Communities were inoculated into the leaf-based medium (ALM) at an initially consistent total cell density, with each member at an equal density reduced in proportion to the richness of the community. We passaged each community into fresh medium (1:100 dilution) every 24 h up until its invasion treatment (see below) and for 7 days following the invasion (Fig. 1A). We previously demonstrated that 7 days is sufficient to allow communities in this system to reach an ecologically stable state [26].

To test the effect of invasion timing on community assembly, we invaded each of these 15 communities with three different bacterial invaders at each of three time points in the community assembly process (Fig. 1A). We chose to work with multiple community contexts and invaders to seek general patterns in the effect of invasion timing, rather than outcomes specific to a certain invader and/or community context. We used a strain of *Pseudomonas poae* (*Pseudomonas*_MEJ082), a strain of *Pseudomonas viridiflava* (*Pseudomonas*_RMX3.1b), and a strain of *Xanthomonas campestris* (*Xanthomonas*_S130) as invaders. For each of these invaders, we were able to demonstrate that by adjusting the “propagule pressure” of the invasion (the initial invader density relative to the estimated density of the invaded community), we could observe variable invasion outcomes (Supplementary Fig. 1, “Determining appropriate invading densities for invaders” methods below).

We assessed three invasion timing treatments that targeted distinct phases of the community dynamics. In the “initial invasion” treatment, invaders were added to the community during initial community assembly (T0), when the assembly process had just begun. In the “early invasion” treatment, invaders were added immediately after the first round of passaging (24 h, T1), which is an especially dynamic point in the assembly process [26]. And in the “late invasion” treatment, invaders were added after 7 days of growth (entailing 6 rounds of passaging, T7), when community dynamics had reached a steady state. In all treatments, after adding invaders, communities were passaged for an additional 7 days. Although the initial invasion treatment is not an “invasion” in that the invaders are initially present, it serves as an important reference in defining a baseline of (i) whether a given invader could persist in each community context and (ii) the final community composition. We used high-throughput sequencing to characterize the composition of these communities by mapping short-reads back to previously assembled reference genomes (“Sequencing and read mapping” below).

### Determining appropriate invading densities for invaders

Given that we aimed to assess the effect of invaders on the composition of invaded communities, we wanted to avoid instances where invader density was too low to impact a community or so high that an invader would dominate every invaded community. To identify an appropriate density for each invading isolate, eight of the communities (#8–15) were invaded with each invader across a range of propagule pressures (0.01%, 0.1%, 1%, 10%, 25%, 100% of estimated invaded community density). These cultures were passaged as described for the main experiment, and invader presence was tracked over time by spot plating 20 μL of each culture from each timepoint onto 1× tryptic soy agar (TSA) plates containing 70 μg/mL gentamicin (Supplementary Fig. 1). The invader isolates had been previously transformed to contain gentamicin-resistance cassettes through mini-Tn7 insertion [28], allowing us to track the presence of the invaders over time. In this way, we identified densities for each invader that produced a variety of invasion outcomes (i.e. failure to establish vs. persistence at variable abundances). These densities were 0.1% of estimated total community density for *X. campestris* and 10% for both *P. poae* and *P. viridiflava*.

### Spent media assays

We performed spent media assays to evaluate the ability of the invaders to use resources unused or produced by the invaded communities. We performed such assays for the early and late invasion treatments. For the early treatment, we isolated spent medium from uninvaded communities after 24 h of growth (immediately prior to passaging and addition of invaders). For the late treatment, we isolated spent medium from uninvaded communities after 7 days of growth (immediately prior to the 7th passage). We isolated spent medium from each community by pelleting the bacterial cells (centrifuged for 10 min at 3000 RCF) and filtering ~150 μL of supernatant through 0.2-μm polytetrafluoroethylene filtration plates (Pall Corporation, Port Washington, NY, USA). The filtrate was then pooled by community (to produce a representative spent medium for a given community, homogenizing variation among replicates) and amended with M9 salts (at a final concentration of 0.3×) to ensure minimal metabolic needs were met and to thus focus on unused/produced sources of carbon. Prior to inoculation, invader stocks were pelleted and washed in 10 mM MgSO_4_ twice to minimize media carryover from the stocks. Each invader was subsequently resuspended in 10 mM MgSO_4_, and 5 μL was inoculated into 200 μL of each spent medium in triplicate and cultured at 28°C in 96-well clear-bottom plates. Negative controls were present in each plate, containing only 10 mM MgSO_4_ buffer and M9 salts (0.3×). These negative controls were used to subtract background growth from the invaders cultured in spent medium. Growth was assessed by optical density (OD600 nm) after 48 h.

### DNA extraction

DNA was extracted from synthetic communities using an enzymatic digestion and bead-based purification. Cell lysis began by adding 250 μL of lysozyme buffer (tris-EDTA buffer (TE) + 100 mM NaCl + 1.4 U/μL lysozyme) to 300 μL of thawed sample and incubating at room temperature for 30 min. Next, 200 μL of proteinase K buffer (TE + 100 mM NaCl + 2% sodium dodecyl sulfate (SDS) + 1 mg/mL proteinase K) was added. This solution was incubated at 55°C for 4 h and mixed by inversion every 30 min. After extraction, the samples were cooled to room temperature before adding 220 μL of 5 M NaCl to precipitate the SDS. The samples were then centrifuged at 3000 RCF for 5 min to pellet the SDS. A Tecan (Männedorf, Switzerland) Freedom Evo liquid handler was used to remove 600 μL of supernatant. The liquid handler was then used to isolate and purify the DNA using SPRI beads prepared as previously described [29]. Briefly, samples were incubated with 200 μL of SPRI beads for 5 min before separation on a magnetic plate, followed by two washes of freshly prepared 70% ethanol. Samples were then resuspended in 50 μL of ultrapure H_2_O, incubated for 5 min, separated on a magnetic plate, and the supernatant was transferred to a clean polymerase chain reaction (PCR) plate. Purified DNA was quantified using a Picogreen assay (ThermoFisher, Waltham, MA, USA) and diluted to 0.5 ng/μL with the aid of a liquid handler.

### Sequencing library preparation

Libraries were prepared using Nextera XT DNA library preparation kits (Illumina, San Diego, CA, USA). Our protocol differed from the published protocol in two ways: (1) the tagmentation reaction was scaled down such that 1 μL of purified DNA, diluted to 0.5 ng/μL, was added to a solution of 1 μL buffer + 0.5 μL tagmentase, and (2) a KAPA HiFi PCR kit (Roche, Basel, Switzerland) was used to perform the amplification in place of the reagents included in the Nextera XT kit. PCR mastermix (per reaction) was composed of 3 μL 5× buffer, 0.45 μL 10 mM dNTPs, 1.5 μL i5/i7 index adapters, 0.3 μL polymerase, and 5.75 μL ultrapure H_2_O. The PCR protocol was performed as follows: 3 min at 72°C; 13 cycles of 95°C for 10 s, 55°C for 30 s, 72°C for 30 s; 5 min at 72°C; hold at 10°C. Sequencing libraries were manually purified by adding 15 μL of SPRI beads and following the previously described approach, eluting into 12 μL of ultrapure H_2_O. Libraries were quantified by Picogreen assay, and a subset of libraries were run on an TapeStation 4200 system (Agilent, Santa Clara, CA, USA) to confirm that the fragment size distributions were of acceptable quality. The libraries were then diluted to a normalized concentration with the aid of a liquid handler and pooled. The pooled libraries were concentrated on a vacuum concentrator prior to size selection for a 300–600-bp range on a Blue Pippin (Sage Science, Beverly, MA, USA). The distribution of size-selected fragments was measured by TapeStation. Size-selected pool libraries were quantified by Picogreen assay and qPCR (KAPA Library Quantification Kit).

### Sequencing and read mapping

We characterized the compositions of our synthetic communities with a shallow metagenomics approach. Samples were sequenced on a NovaSeq 6000 System (Illumina). Reads were quality filtered and adapter/phiX sequences were removed using BBDuk from the BBTools suite [30]. To address ambiguously mapped reads resulting from genomic similarity between some closely related isolates, reads were mapped to reference genomes using Seal (BBTools) twice, once with the “ambig” flag set to “toss” (where ambiguously mapped reads were left out) and once with the “ambig” flag set to “random” (where ambiguously mapped reads were randomly distributed to equally likely references). By comparing the results between these two strategies, we identified sets of reference genomes that resulted in high numbers of ambiguous reads (due to similarity). We corrected for this ambiguity by identifying the number of reads that were removed in the “toss” setting (i.e. the difference in mapped reads between the “toss” and “random” settings) and reallocating those reads based on the proportion of reads unambiguously mapped to each isolate in the “toss” setting (as those proportions represents our best estimate of the true relative abundances of similar isolates). To avoid mischaracterizing the composition of our synthetic communities due to contamination or nonspecific mapping, isolates with <1% of total mapped reads for a given sample were ignored.

### PERMANOVA analysis

Single-factor permutational multivariate analysis of variance (PERMANOVA) tests were used to determine if the shifts in community composition resulting from the invasion timing treatments were distinct from the uninvaded control communities. Tests were performed using the “adonis2” function from the R package “vegan” (v2.6-4, ref. [31]). Bray–Curtis dissimilarity was used to measure the compositional effect of a given treatment. All tests were performed with 999 permutations and the permutations were blocked by community identity. Principal coordinate analysis was performed with the R package “phyloseq” (v1.32.0, ref [32]).

### Statistical analysis and data visualization

Statistical analysis and figure generation was performed in R v4.0.2 (ref. [33]) with aid from the following packages: tidyverse v1.3.0 (ref. [34]), reshape2 v1.4.4 (ref. [35]), car v3.0-11 (ref. [36]), vcd v1.4-11 (ref. [37]), margins v0.3.26 (ref. [38]), broom.mixed v0.2.9.5 (ref. [39]), and lme4 v1.1-26 (ref. [40]). All scripts are provided in the supplementary materials. Multiple-sequence alignment for phylogenetic distance analysis was performed using the Clustal Omega algorithm available through the EMBL-EBI bioinformatics job dispatcher [41].

## Results

### Invasions were commonly unsuccessful, and more so in high-richness communities

We define a successful invasion as one in which an invader persisted in the community to which they were introduced, regardless of the invader’s relative abundance. In general, successful invasions were uncommon (Table 1). Across all invaders and all communities, successful invasion was observed ~24% of the time. In these instances of successful invasion, the presence of the invader resulted in the exclusion of at least one resident community member 46% of the time. Some invaders were more successful than others (Chi-square test of independence: *P* value <2e^−9^, Fig. 1B), with *X. campestris* displaying the highest success rate at 39%, *P. viridiflava* showing the lowest at 6%, and *P. poae* exhibiting an intermediate rate of 27% (Table 1A). The most successful invader displayed the lowest mean relative abundance (0.03 ± 0.02), whereas the less successful invaders were more likely to attain a higher relative abundance (mean relative abundance for *P. viridiflava* and *P. poae* were 0.27 ± 0.12 and 0.16 ± 0.18, Table 1A).

**Table 1:** Summary of invasion outcomes by (A) invader and (B) community.

(A) Invader	Successful invasions	Unsuccessful invasions	Success rate	Mean relative abundance (±SD)
*Pseudomonas poae*	37	98	0.27	0.16 ± 0.18
*Pseudomonas viridiflava*	8	127	0.06	0.27 ± 0.12
*Xanthomonas campestris*	49	78	0.39	0.03 ± 0.02
**(B) Community**	**Initial community richness**	**Mean early richness**	**Mean final richness**	**Success rate**
01	8	4	2	0.31
02	8	6.6	4.4	0.12
03	8	4	4	0.54
04	8	4.3	2.9	0.54
05	8	7	4.8	0.38
06	8	6.7	3.7	0.54
07	16	8.2	5.3	0.12
08	16	8.8	4.6	0.12
09	16	5.8	5.8	0.22
10	16	9.4	4.7	0.19
11	24	10.4	5.2	0.07
12	24	10.7	6.5	0.22
13	24	9.3	4.6	0
14	32	9.8	7.3	0.22
15	48	12.8	6.7	0

In B, “initial community richness” represents the number of strains initially present, excluding the invader, whereas “mean early richness” and “mean final richness” represent the mean richness observed among replicates in the respective uninvaded control communities. Success rate is calculated as the number of successful invasions out of the total number of invasions.

**Figure 2:**
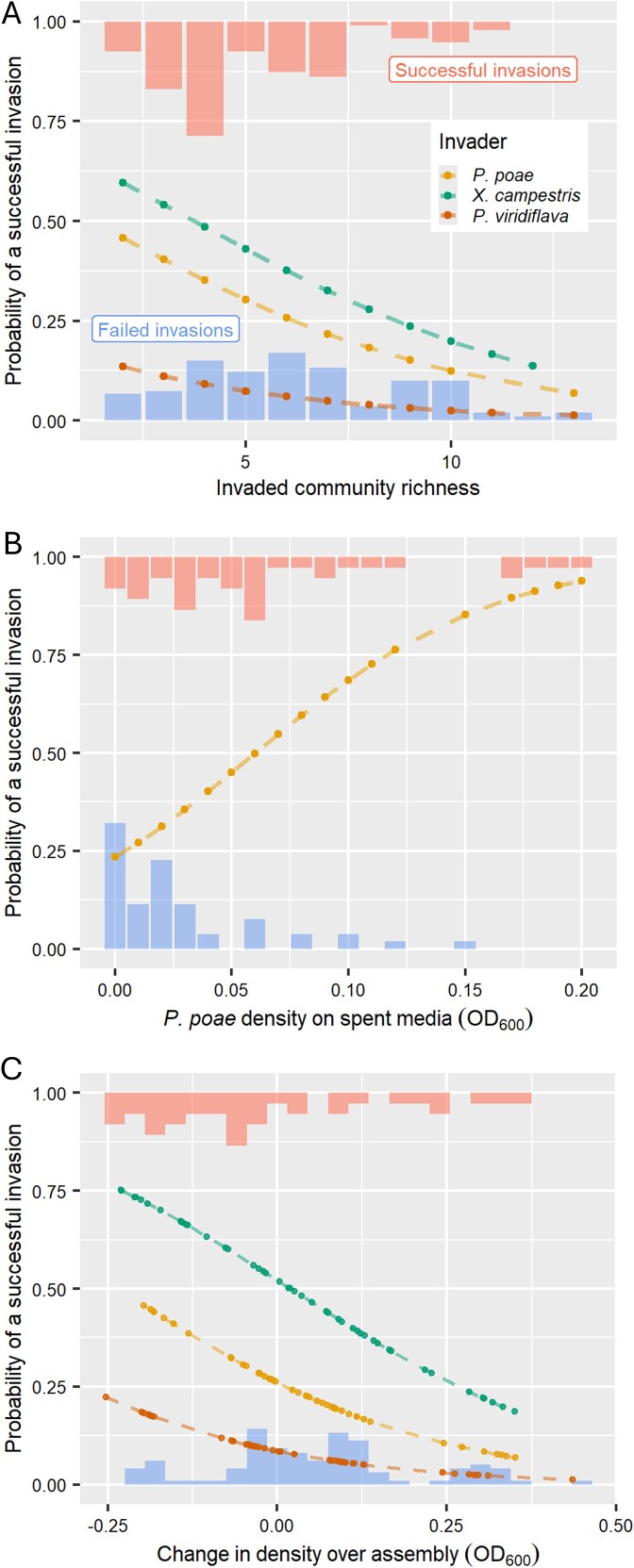
Invaded community richness and estimates of community resource use efficiency were predictive of invasion outcome: plots depicting the results of logistic regressions analyzing the relationship between invasion outcome (success/failure) with (A) the richness of an invaded community measured immediately prior to invasion, (B) the growth (optical density) of the invader *Pseudomonas poae* (MEJ082) on spent media, and (C) the change in density between day 1 and day 6 of the “late invasion” communities prior to invasion. Proportional histograms at the top and bottom of each plot represent the observed distributions of invaded community richness, density, or change in density, partitioned by invasion outcome (bottom = failed invasion, top = successful invasion). The dotted lines represent the fitted relationship of each model for each invader considered in the analysis.

We also observed an association between invaded community and invasion success, with communities 3, 4, and 6 experiencing high invasion success rates across invaders (Table 1B, Fig. 1C). Those three communities were among the communities with the lowest initial and final richness. Given that our communities varied in richness across invasion treatments, we used mixed-effect logistic regression to analyze the relationship between invasion success or failure and richness of an invaded community, taking into account the identity of the invader as a random effect. We found that the probability of successful invasion decreased as the richness of communities immediately prior to invasion increased, but with a low average marginal effect; an increase in richness of one was associated with a 3.5% reduction in the probability of an introduction leading to a successful invasion (Fig. 2A, Supplementary Table 2). The significance of this negative relationship persisted for two of the three invaders when considered independently (*P* values: *P. poae*—.66, *P. viridiflava*—0.006, *X. campestris*—<8e^−5^).

We additionally investigated if other measures of community diversity were significantly associated with invasion outcome. First, we asked if there was a relationship between invasion outcome and community diversity, as measured by Simpson’s Diversity Index or Pielou’s Evenness Index, through mixed-effect logistic regressions considering invader identity as a random effect. We failed to observe significant relationships for either metric (*P* values .11 and .8, respectively, Supplementary Fig. 2). Next, we asked if invasion outcome was related to phylogenetic similarity, both within an invaded community and relative to the invader. To assess this, we calculated the phylogenetic distance between all strains used in the experiment from a multiple-sequence alignment of the DNA gyrase subunit B gene (*gyrB*), a commonly used and informative phylogenetic marker [42]. We performed mixed-effect logistic regression (with invader identity as a random effect) to evaluate the relationships between invasion outcome and either average phylogenetic distance between members of the invaded community or average phylogenetic distance between the invader and the invaded community. For both analyses, we used the composition of the invaded community immediately prior to the introduction of the invader. We failed to observe a significant relationship between the average distance among community members and invasion outcome (*P* value .083, Supplementary Fig. 2c). However, we did observe statistical support for a positive relationship between invasion success and average phylogenetic distance to the invader (coefficient 0.041, SE 0.019, *z*-value 2.1, *P* value .031, Supplementary Fig. 2d).

The presence of a phylogenetic effect suggested community composition was relevant to invasion outcome. To further interrogate this compositional effect, we performed a series of Fisher’s exact tests to ask if the presence or absence of specific community members was associated with the invasion outcomes of each invader. After performing multiple test correction using the Benjamini–Hochberg procedure, we observed five marginally significant associations between community members and invasion outcomes (Supplementary Table 3). Four of the five significant associations were negative, where the presence of a community member was associated with a lower likelihood of invasion. Strains closely related to an invader could have both positive and negative associations with that invader, as demonstrated by the positive relationship between *Pseudomonas*_S91 and *P. poae* but the negative relationship between *Pseudomonas*_S105 and *P. poae*.

### Resource use efficiency was associated with invasion success

If invaders are more successful when introduced into communities with an abundance of metabolites suitable for cross-feeding, then we would expect invasion success to be related to low resource use efficiency. To examine this possibility, we evaluated how well invaders were able to grow on the spent media of a community prior to invasion (methods) and tested whether growth on spent media was predictive of invasion outcome. Briefly, we filtered each of the communities after 1 day and 7 days of growth (representing the communities immediately prior to the early-invasion and late-invasion treatments, respectively) to remove bacterial cells and isolate sterile spent medium. We then assessed the growth of each of the three invader species when cultured on each spent medium, measuring OD600 after 48 h.

Extensive growth was rare and generally restricted to *P. poae* (Supplementary Fig. 3a), which was the invader with an intermediate probability of invasion success at 27%. Although there was insufficient variation in invader growth to explore how community composition influenced the growth of *P. viridiflava* and *X. campestris*, we used logistic regression to analyze the relationship between growth of *P. poae* on spent media from the 15 communities and whether an invasion was successful. We found that a 0.1 increase in *P. poae* optical density was associated with a ~40% average increase in the probability of a successful invasion (Supplementary Table 2, Fig. 2B). Thus, for *P. poae*, invasion success was positively associated with its ability to use the resources unused or produced by a given community. To ask if this relationship was related to community richness, we calculated the correlation between an invader’s growth on spent media and the richness of the community from which that media came. We observed a modest negative relationship (Pearson correlation coefficient—0.12, *P* value .047, Supplementary Fig. 3b).

We additionally wanted to assess if a change in resource use efficiency over the course of community assembly was associated with invasion outcome. To approximate a change in resource use efficiency, we compared the initial (T1) and late stage (T6) densities (OD600) of each uninvaded community. Generally, community density modestly increased over this period, with a statistically significant average increase in optical density of 0.035 (one-sample *t*-test: *P* value .01). However, there was variation in changes in density, with some communities increasing and others decreasing (Fig. 2C). We used mixed-effect logistic regression to relate these changes in density over time with invasion outcome in the late-invasion treatment, taking into account the identity of the invader as a random effect. We observed a significant negative relationship between an increase in density and the probability of successful invasion; namely, a decrease in density of 0.1 during assembly (in the absence of an invader) was associated with a ~7% increase in the probability of a successful invasion (Supplementary Table 2, Fig. 2C). This did not simply reflect that less dense communities were more prone to invasion but rather that communities that declined most extensively during pre-invasion assembly were more vulnerable to invasion; this was clear because pre-invasion density itself was not significantly associated with invasion success by logistic regression (*P* value .52). Additionally, we asked if the relationship between change in community density during pre-invasion assembly and invasion success was related to richness by testing for a correlation between community richness prior to invasion and change in density over assembly. We observed a positive relationship, indicating that communities that maintained higher richness were more likely to increase in density (Pearson correlation coefficient 0.37, *P* value <9e^−6^, Supplementary Fig. 3c).

### Invasion timing affected invasion outcome and effect on community composition

**Figure 3:**
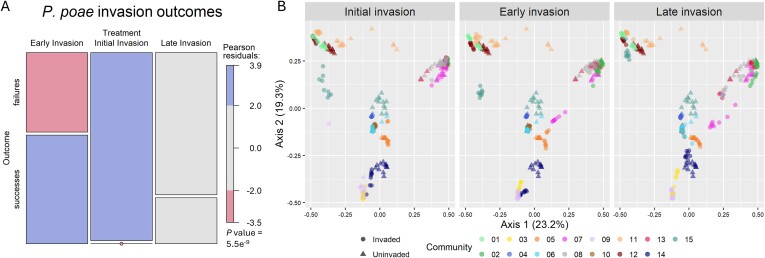
Invasion timing affected invasion outcome and compositional effect: (A) mosaic plot representing the relative frequencies of invasion outcomes across the three invasion timing treatments for invasions by *P. poae*. Invasion outcome was defined as a “success” (bottom box) if the invader persisted over time or a “failure” (top box) if the invader was ultimately excluded. Chi-square test of independence demonstrates a significant association between invasion outcome and invasion timing (*P* value <6e^−9^), with the Pearson residuals indicating invasion success was significantly over-represented in the early-invasion treatment whereas invasion failure was significantly over-represented in the initial-invasion treatment (significantly over- or under-represented outcomes are highlighted). The late invasion results did not significantly deviate from the null expectations. Pearson residuals >2 or <−2 indicate a count value >2 SD from the expectation. (B) Bray–Curtis dissimilarity–based principal coordinate ordinations of communities from each invasion treatment alongside the uninvaded reference communities for comparison. Communities are identified by color; invasion status of the community (uninvaded/invaded) is represented by point shape.

To assess the effect of the timing of an invasion on its outcome, we first compared the probability of a successful invasion across invasion treatments. We observed that there was a significant association between invasion timing and invasion outcome for only a single invader, *P. poae* (Chi-square test of independence, *P* value <6e^−9^). For that invader, successful invasions were statistically more likely to succeed in the early-invasion treatment and fail in the initial-invasion treatment (Fig. 3A). Although we only observed this effect for a single invader (Supplementary Fig. 4), we wondered if we might observe a more general effect of invasion timing when considering the impact of an invasion measured as a shift in the composition of the community, rather than as a binary outcome (success/failure).

To test whether invasion timing had an impact on the composition of communities, we calculated the mean Bray–Curtis dissimilarity of each invaded community relative to the respective uninvaded control community (across replicates within each timing treatment), disregarding the invader if it was present (Fig. 3B, Supplementary Table 4a). We found that the early-invasion treatment, on average, showed the greatest dissimilarity (0.457 ± 0.21) followed by the late-invasion (0.401 ± 0.19) and initial-invasion (0.375 ± 0.22) communities. We then used pairwise single-factor PERMANOVA tests to determine if the invasion treatments resulted in community compositions distinct from the uninvaded controls (methods). We observed that all invasion timing treatments resulted in significantly distinct community compositions (all *P* values ≤.003 after multiple testing correction). We next sought to determine if the invasion timing treatments differed in the extent to which they shifted the composition of the invaded communities. A one-way ANOVA found that the mean Bray–Curtis dissimilarity differed between the invasion treatments (*F*_2,381_ = 5.2, *P* value .006, Supplementary Table 4b). This result was general across invaders, as inclusion of invader identity as an interaction effect was not significant (*P* value .95) and made no change to the effect of invasion timing. *Post hoc* tests showed strong support for a significant difference in dissimilarity between the early- and initial-invasion treatments with an average difference of 0.08 (Tukey’s honest significant test: 95% CI [0.02, 0.14], *P* value = .005, Supplementary Table 4c). There was also marginal support for a difference between the early- and late-invasion treatments (Tukey’s honest significant test: 95% CI [−0.005, 0.12], *P* value = .081, Supplementary Table 4c).

Given that we had previously identified associations between invasion outcome and both community richness and invader growth on spent media, and that these factors may change over the course of community assembly, we included these variables as covariates in additional analyses (analysis of covariance (ANCOVA)) to determine if they could explain the effect of invasion timing (Supplementary Table 5). Controlling for differences in richness had little impact on the significance of invasion timing in these models, suggesting that this factor could not explain the effect of timing (Supplementary Table 5b). However, invader growth on spent media was a significant covariate that reduced the effect of invasion timing (Supplementary Table 5c). This analysis, however, only considered the early- and late-invasion treatments, as those were the only treatments for which we could measure growth on spent media prior to invasion.

### Transient invasions had persistent effects on community composition

**Figure 4:**
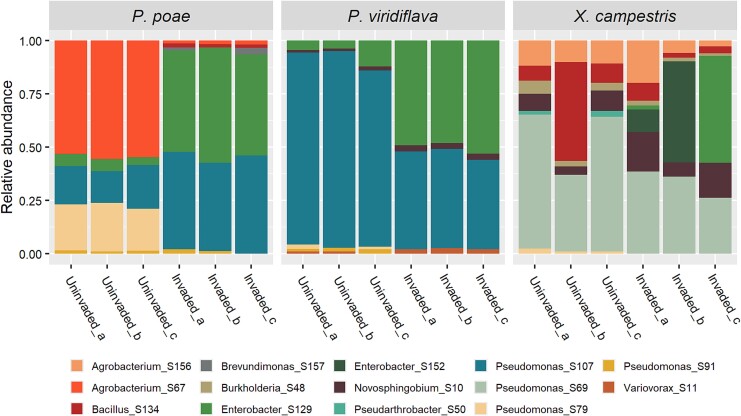
Transient invasions could have large and long-lasting effects on community composition: examples of instances where each invader (*P. poae*, *P. viridiflava*, and *X. campestris*, respectively) had a strong and lasting effect on the composition of the invaded community despite not persisting in the community over time.

Given that many invasions ultimately failed, we wanted to identify how these transient invasions could affect the composition of the communities that excluded them. We observed that, across all invaders, transient invasions could have profound and persistent effects on final community composition (Fig. 4). Furthermore, 50% of transient invasions resulted in the loss of at least one member from the invaded community. These results demonstrate that even failed invasions could have an ecologically relevant effect.

## Discussion

We hypothesized that the timing of an invasion would impact its outcome because factors affecting ecological invasions change over the course of community assembly. Community diversity is one such dynamic factor. Indeed, the relationship between diversity and community invasibility has long been studied in plant [15, 17, 20, 43, 44, 45] and experimental bacterial communities [13, 16, 18], as well as in the context of enteric pathogens [45, 46]. Work in multiple biological systems has shown that invasion is less successful in diverse communities [13, 15, 16, 18]. This phenomenon may be explicable because increased diversity can result in more complete occupancy of available niche space, thus increasing community resource use efficiency and reducing resources that are available for an invader [13, 20, 47]. Our results are in general alignment with this effect, as lower richness communities were more likely to be successfully invaded (Fig. 2A). We did not observe significant relationships between invasion outcome and other metrics of community diversity (Simpson’s Diversity or Pielou’s Evenness Indexes). Our findings regarding richness, however, suggest that the relationship we observed between richness and invasion outcome is connected to a relationship between community resource use and richness. For the invader *P. poae*, growth on spent medium was positively associated with invasion success (Fig. 2B) and negatively correlated with community richness (Supplementary Fig. 3b). Furthermore, for the late-invasion treatment, the change in community density over the course of pre-invasion assembly was positively correlated with pre-invasion richness (Supplementary Fig. 3c). These observations point toward a positive relationship between richness and productivity that is relevant to invasion outcome. Finally, the observed effect of invasion timing was reduced when we incorporated invader growth on spent media (Supplementary Table 5c), suggesting that the impact of invasion on community composition was, at least in part, modulated by available resources. Altogether, these findings support the hypothesis that richness, at least in part, can affect the outcome of an invasion by affecting the resource use of an invaded community.

Another factor relevant to invasion outcome is community composition, which inherently changes as community members are filtered out during assembly. This is relevant to invasibility in that invaders can be excluded if they compete with species that share similar nutrient requirements [48, 49, 50]. This mechanism, referred to as the sampling effect, is also related to richness, as higher richness increases the chance that a community contains species capable of excluding an invader through competition [20, 22, 23, 51]. Indeed, we observed that lower richness communities were generally more susceptible to invasion (Fig. 2A), but that susceptibility also varied among low richness communities (Table 1B), suggesting the sampling effect was present within our experiment. The highly dynamic nature of microbial community assembly made it possible for us to compare invasions during transiently dynamic versus more stable time points but raise challenges for interrogating the effects of specific community members on invasion outcome. Despite these challenges, we found evidence supporting the effect of community composition and species identity on invasion outcome. We observed that the presence/absence of five community members was significantly associated with the outcomes of invasions by individual invaders (Supplementary Table 3). Additionally, we observed a modest but significant positive relationship between successful invasion and average phylogenetic distance between the invader and members of the invaded community. This suggests that communities containing members more closely related to an invader, and thus potentially more likely to share overlapping metabolic niches [52], are on average less likely to be invaded by related invaders. Nevertheless, specific strain identity was relevant even among closely related strains, as we observed that the success of *P. poae* was both facilitated and hindered by the presence of closely related pseudomonads (Supplementary Table 3). Thus, the effect of richness on invasion outcomes appears to have also been mediated by community composition through the sampling effect, potentially through both changing competition over a shared metabolic niche or through other strain specific effects.

We can use the effects described above to interpret the differences we observed between invasion timing treatments, namely, why the early invasions appeared most impacted by invasion (Fig. 3, Supplementary Table 4). First, the initial-invasion treatment was potentially more resistant to invasion because the increased richness encountered by invaders in this treatment (as no competitive exclusion could yet have occurred) increased the chances that a community member could suppress or exclude the invader, thus decreasing the chance of an impactful or successful invasion through the sampling effect. This aligns with the fact that we only saw this effect on invasion outcome for one invader, *P. poae* (Fig. 3A), as this mechanism is dependent on invader identity/community composition, and thus potentially more variable across invaders. As for the late-invasion treatment, the enhanced resistance to invasion in that treatment was associated with decreased invader growth on spent media (Supplementary Table 5c). One contributing factor to the decrease in resources available in the spent media was likely the increase in community density (Fig. 2C). Communities may have additionally achieved higher resource use efficiency over time through mechanisms such as changes in metabolic regulation or evolution that resulted in community members becoming better able to utilize available resources [53, 54, 55, 56, 57]. Both mechanisms align with our observation that an increase in community density over the course of assembly was associated with decreased invasibility and could thus contribute to the moderate decrease in invasibility of communities later in the assembly process.

The mechanisms observed as relevant in the initial- and late-invasion treatments can lead to a hypothesis as to why communities in the early treatment were most impacted by invasion. We hypothesize that, relative to the initial-invasion communities, the rapid loss in richness observed within the first 24 h of the experiment reduced the protective benefit of the sampling effect in the early-invasion communities (Table 1b). And relative to the late-invasion communities, the early-invasion communities did not have an opportunity to increase in community density/resource use efficiency over the course of assembly and thus increase resistance to invasion through that mechanism. In other words, the uniquely transient state of the early-invasion communities made them doubly impaired in their capacity to resist the effects of invasion through the mechanisms we observed to be most relevant in our system.

Consistent with our results, Rivett *et al*. [58] reported decreased success of invasions later in the assembly process and identified change in resource availability across community assembly as a mechanism underlying invasion outcome. In that study, assembly occurred in a static environment with no nutrient replenishment. Our method of assembly through passaging represents a distinct assembly process akin to environments with higher sustained metabolic activity resulting from periodic influxes of resources (e.g. the gut environment). Despite the difference in nutrient dynamics between our two systems, the convergence of our results suggests the relationship between invasion timing and outcome is robust across environments.

Previous work investigating the importance of invasion timing has focused on the synchronization of invasions with periods of increased resource availability [22, 59]. This is also related to work investigating the relationship between disturbance and invasibility, which has posited that disruptions in community resource use efficiency enhances the opportunity for invasion [60, 61, 62]. Our results are in general alignment with these perspectives, as they rely on periods of increased relative resource availability as predictors of invasion outcome. However, in contrast to past work, our results stem from the natural, dynamical properties of community assembly rather than extrinsic perturbations. Here, we demonstrate that transient dynamics, whether caused through ecological perturbation or the natural assembly process, facilitate invasion.

We observed that even unsuccessful invasions could have large effects on the final composition of the communities from which they were excluded (Fig. 4). It has been previously observed that transient invasions, by individual species or low-density communities, can cause profound compositional and functional shifts in both simple synthetic communities as well as complex soil communities [63, 64, 65]. Our work furthers those findings by demonstrating that the extent of such community-level shifts can be associated with the state of community assembly at the time of invasion. It has also been shown that minor differences in transient states early in the assembly of bacterial communities can lead to divergent final community compositions [53]. This context can help us understand why the initial- and early-invasion treatments could lead to distinct final community compositions (Fig. 3B, Supplementary Table 4) as the communities at T0 and T1 were sufficiently different that it is unsurprising that disruption via invasion drove divergent paths of further assembly. Broadly, our findings also relate to priority effects, the well-established phenomenon of historical contingency in community assembly [66, 67]. From this ecological perspective, our work can be viewed as a study of how the magnitude of priority effects varies over the course of assembly and the underlying mechanisms contributing to such variation.

Overall, we show that invasion of a synthetic bacterial community at different points of the community assembly process affects invasion success and the impact of the invaders on the resident community. We found that invasions that occurred during a particularly transient phase of the community assembly process had an increased impact on community composition, and in one case, an increased invasion success rate. These findings align with the expected effects of decreased diversity on resource use efficiency and the sampling effect. We also found strong effects of introduced species that failed to persist on community composition. In summary, our findings demonstrate that the resilience of microbial communities to the effects of invasion varies over the course of community assembly and can be explained by known mechanisms from invasion ecology. These results further our understanding of the factors affecting the invasibility of microbial communities, with implications relevant to human health, agriculture, and industry.

## Supplementary Material

Supplementary_wrae220

## Data Availability

The datasets generated during and/or analyzed during the current study are available in the NCBI Whole Genome and Sequence Read Archive repositories (accessions: PRJNA953780, PRJNA1073231).
