# Supplementary material for: Impact of timing on the invasion of synthetic bacterial communities
Source: ISME J. 2024 Nov 5;18(1):wrae220. doi: 10.1093/ismejo/wrae220 (PMC11605641; doi:10.1093/ismejo/wrae220)
Supplement: Supplementary_wrae220 [file supplementary_wrae220.zip › Supplementary_wrae220/Supplementary tables 2 to 5.pptx]

## Slide 1
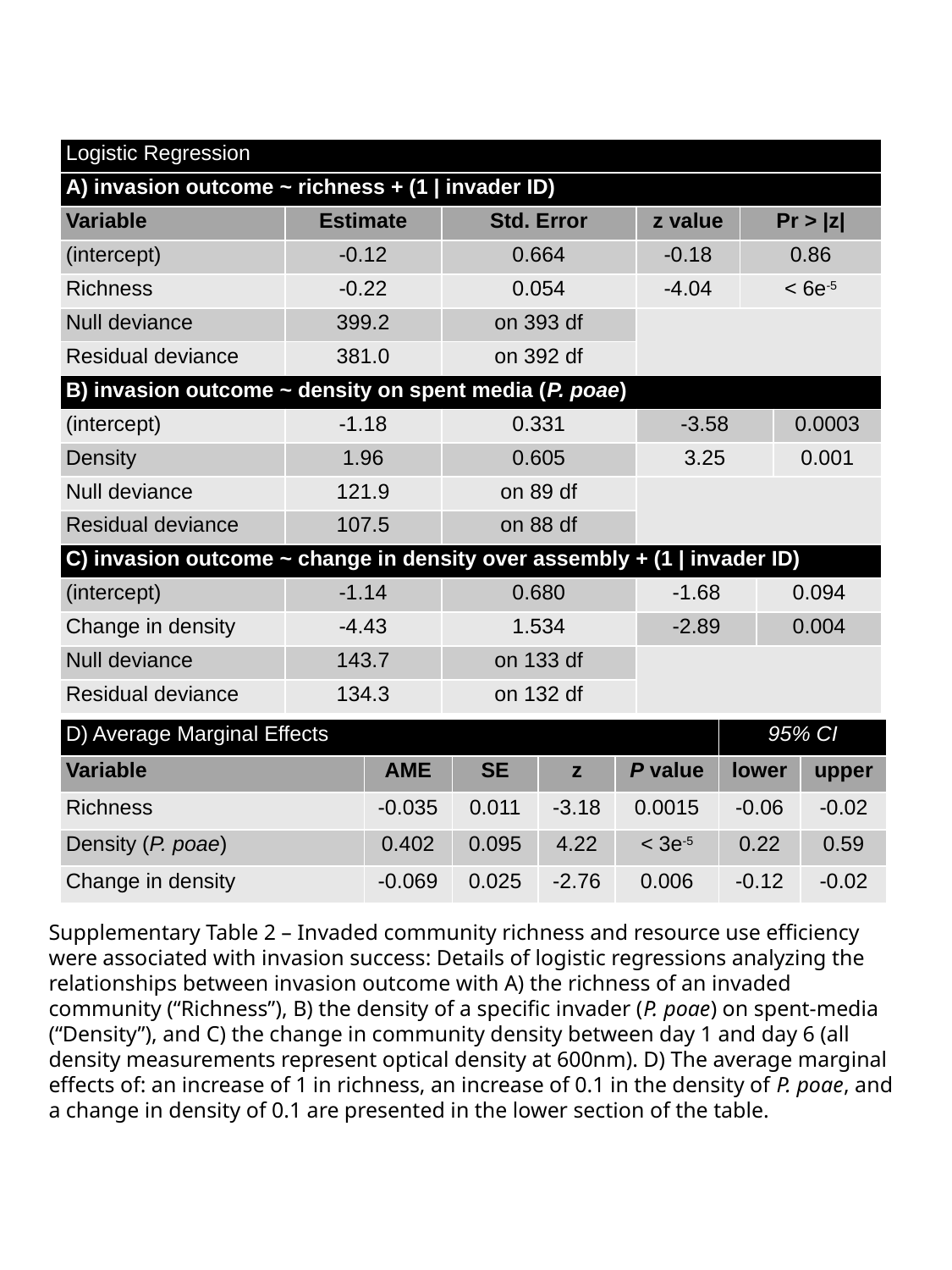

| Logistic Regression | | | | | | |
| --- | --- | --- | --- | --- | --- | --- |
| A) invasion outcome ~ richness + (1 | invader ID) | | | | | | |
| Variable | Estimate | Std. Error | z value | Pr > |z| | | |
| (intercept) | -0.12 | 0.664 | -0.18 | 0.86 | | |
| Richness | -0.22 | 0.054 | -4.04 | < 6e-5 | | |
| Null deviance | 399.2 | on 393 df | | | | |
| Residual deviance | 381.0 | on 392 df | | | | |
| B) invasion outcome ~ density on spent media (P. poae) | | | | | | |
| (intercept) | -1.18 | 0.331 | -3.58 | | | 0.0003 |
| Density | 1.96 | 0.605 | 3.25 | | | 0.001 |
| Null deviance | 121.9 | on 89 df | | | | |
| Residual deviance | 107.5 | on 88 df | | | | |
| C) invasion outcome ~ change in density over assembly + (1 | invader ID) | | | | | | |
| (intercept) | -1.14 | 0.680 | -1.68 | | 0.094 | |
| Change in density | -4.43 | 1.534 | -2.89 | | 0.004 | |
| Null deviance | 143.7 | on 133 df | | | | |
| Residual deviance | 134.3 | on 132 df | | | | |
| D) Average Marginal Effects | | | | | 95% CI | |
| --- | --- | --- | --- | --- | --- | --- |
| Variable | AME | SE | z | P value | lower | upper |
| Richness | -0.035 | 0.011 | -3.18 | 0.0015 | -0.06 | -0.02 |
| Density (P. poae) | 0.402 | 0.095 | 4.22 | < 3e-5 | 0.22 | 0.59 |
| Change in density | -0.069 | 0.025 | -2.76 | 0.006 | -0.12 | -0.02 |
Supplementary Table 2 – Invaded community richness and resource use efficiency were associated with invasion success: Details of logistic regressions analyzing the relationships between invasion outcome with A) the richness of an invaded community (“Richness”), B) the density of a specific invader (P. poae) on spent-media (“Density”), and C) the change in community density between day 1 and day 6 (all density measurements represent optical density at 600nm). D) The average marginal effects of: an increase of 1 in richness, an increase of 0.1 in the density of P. poae, and a change in density of 0.1 are presented in the lower section of the table.

## Slide 2
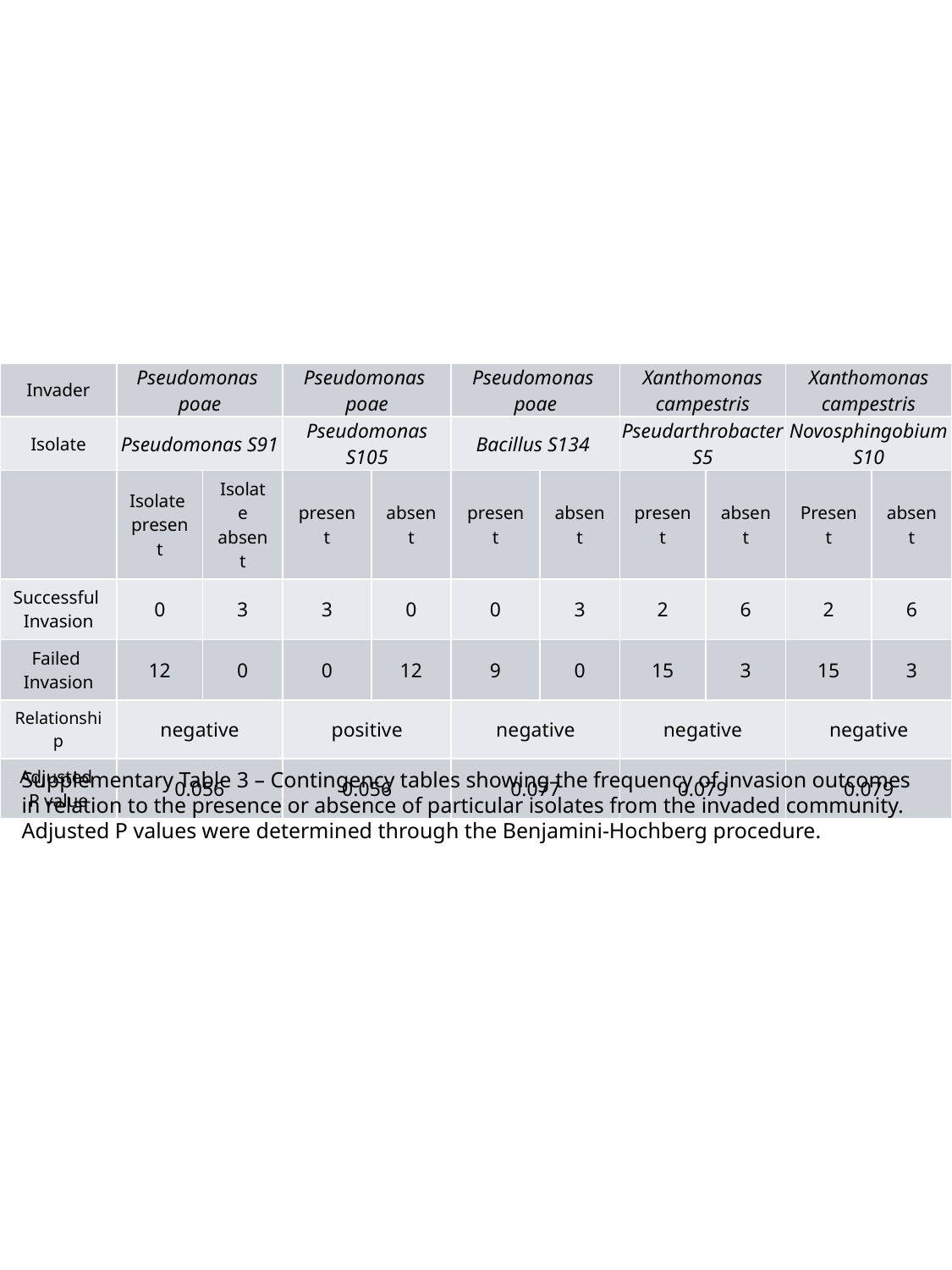

| Invader | Pseudomonas poae | | Pseudomonas poae | | Pseudomonas poae | | Xanthomonas campestris | | Xanthomonas campestris | |
| --- | --- | --- | --- | --- | --- | --- | --- | --- | --- | --- |
| Isolate | Pseudomonas S91 | | Pseudomonas S105 | | Bacillus S134 | | Pseudarthrobacter S5 | | Novosphingobium S10 | |
| | Isolate present | Isolate absent | present | absent | present | absent | present | absent | Present | absent |
| Successful Invasion | 0 | 3 | 3 | 0 | 0 | 3 | 2 | 6 | 2 | 6 |
| Failed Invasion | 12 | 0 | 0 | 12 | 9 | 0 | 15 | 3 | 15 | 3 |
| Relationship | negative | | positive | | negative | | negative | | negative | |
| Adjusted P value | 0.056 | | 0.056 | | 0.077 | | 0.079 | | 0.079 | |
Supplementary Table 3 – Contingency tables showing the frequency of invasion outcomes in relation to the presence or absence of particular isolates from the invaded community. Adjusted P values were determined through the Benjamini-Hochberg procedure.

## Slide 3
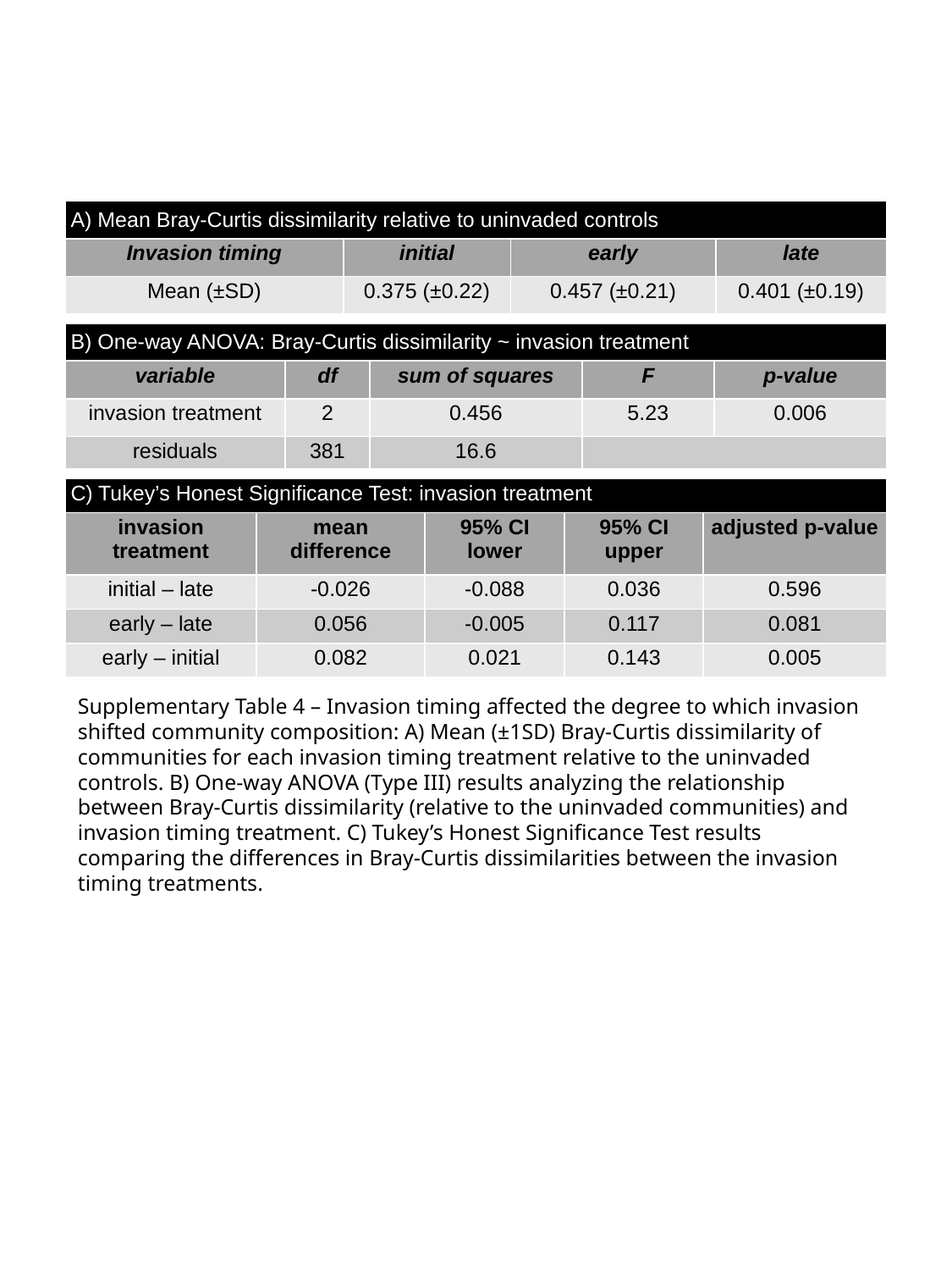

| A) Mean Bray-Curtis dissimilarity relative to uninvaded controls | | | |
| --- | --- | --- | --- |
| Invasion timing | initial | early | late |
| Mean (±SD) | 0.375 (±0.22) | 0.457 (±0.21) | 0.401 (±0.19) |
| B) One-way ANOVA: Bray-Curtis dissimilarity ~ invasion treatment | | | | |
| --- | --- | --- | --- | --- |
| variable | df | sum of squares | F | p-value |
| invasion treatment | 2 | 0.456 | 5.23 | 0.006 |
| residuals | 381 | 16.6 | | |
| C) Tukey’s Honest Significance Test: invasion treatment | | | | |
| --- | --- | --- | --- | --- |
| invasion treatment | mean difference | 95% CI lower | 95% CI upper | adjusted p-value |
| initial – late | -0.026 | -0.088 | 0.036 | 0.596 |
| early – late | 0.056 | -0.005 | 0.117 | 0.081 |
| early – initial | 0.082 | 0.021 | 0.143 | 0.005 |
Supplementary Table 4 – Invasion timing affected the degree to which invasion shifted community composition: A) Mean (±1SD) Bray-Curtis dissimilarity of communities for each invasion timing treatment relative to the uninvaded controls. B) One-way ANOVA (Type III) results analyzing the relationship between Bray-Curtis dissimilarity (relative to the uninvaded communities) and invasion timing treatment. C) Tukey’s Honest Significance Test results comparing the differences in Bray-Curtis dissimilarities between the invasion timing treatments.

## Slide 4
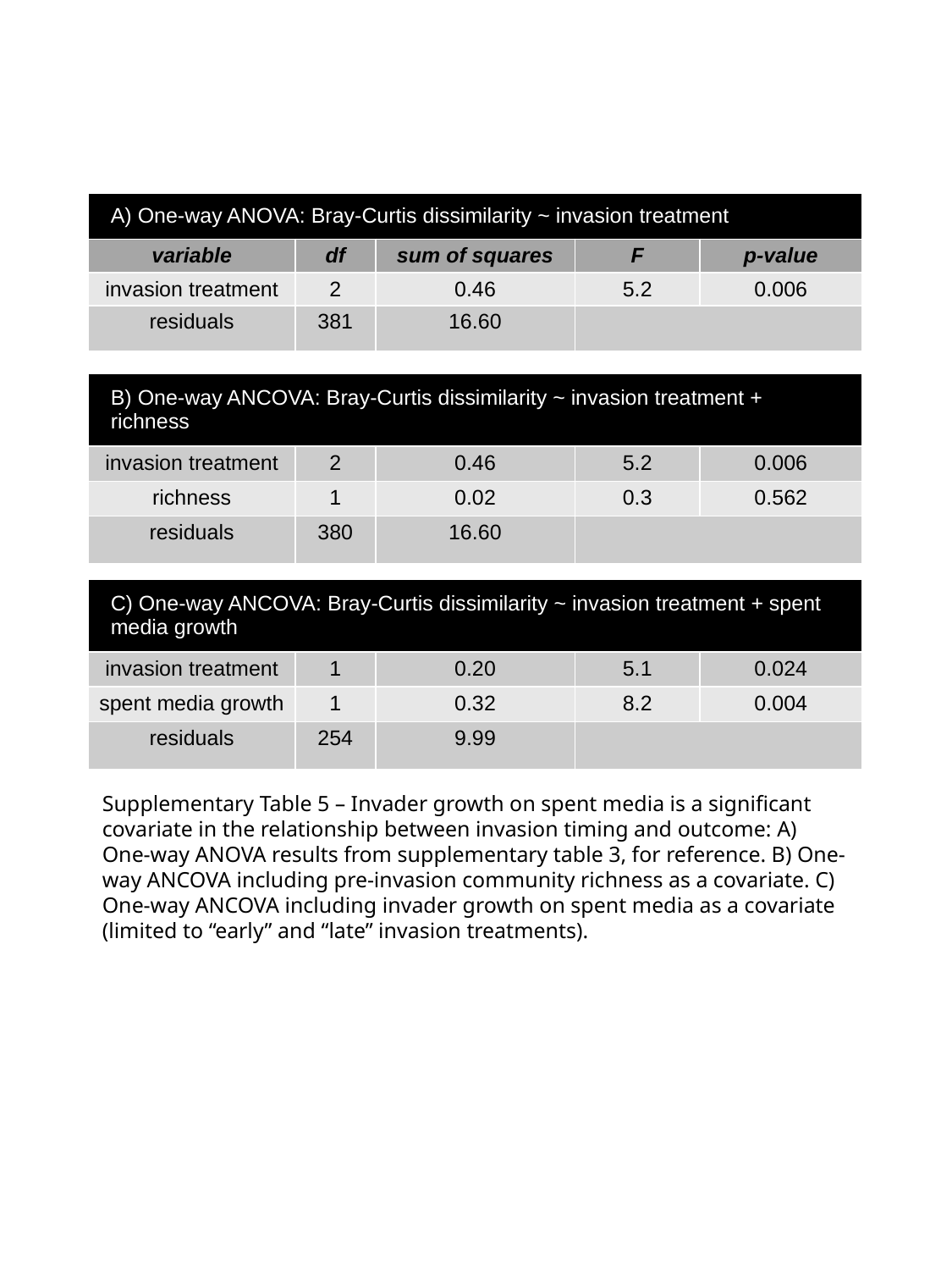

| A) One-way ANOVA: Bray-Curtis dissimilarity ~ invasion treatment | | | | |
| --- | --- | --- | --- | --- |
| variable | df | sum of squares | F | p-value |
| invasion treatment | 2 | 0.46 | 5.2 | 0.006 |
| residuals | 381 | 16.60 | | |
| B) One-way ANCOVA: Bray-Curtis dissimilarity ~ invasion treatment + richness | | | | |
| --- | --- | --- | --- | --- |
| invasion treatment | 2 | 0.46 | 5.2 | 0.006 |
| richness | 1 | 0.02 | 0.3 | 0.562 |
| residuals | 380 | 16.60 | | |
| C) One-way ANCOVA: Bray-Curtis dissimilarity ~ invasion treatment + spent media growth | | | | |
| --- | --- | --- | --- | --- |
| invasion treatment | 1 | 0.20 | 5.1 | 0.024 |
| spent media growth | 1 | 0.32 | 8.2 | 0.004 |
| residuals | 254 | 9.99 | | |
Supplementary Table 5 – Invader growth on spent media is a significant covariate in the relationship between invasion timing and outcome: A) One-way ANOVA results from supplementary table 3, for reference. B) One-way ANCOVA including pre-invasion community richness as a covariate. C) One-way ANCOVA including invader growth on spent media as a covariate (limited to “early” and “late” invasion treatments).
